# Patients’ preferences and perceptions of point-of-care technologies: Survey results from the lung disease community

**DOI:** 10.1017/cts.2026.10791

**Published:** 2026-07-13

**Authors:** Bernard Ofosuhene, Heaven Tatere, Miraf Yeshaneh Tatere, Taylor Orwig, Ziyue Wang, Apurv Soni, Craig M. Lilly, Stephen Anders, Denise Dunlap, Honghuang Lin, Allan Walkey, Chiara Ghezzi, Mary Ann Picard, Varun Ayyaswami, Bryan Buchholz, David D. McManus, Nathaniel Hafer

**Affiliations:** 1 UMass Chan Medical School, Worcester, MA, USA; 2 University of Massachusetts Lowell, Lowell, MA, USA

**Keywords:** Point-of-care test, respiratory diseases, covid-19 pandemic

## Abstract

**Background:**

Point-of-care technology (POCT) has expanded rapidly, particularly since the COVID-19 pandemic. However, less is known about which POCT applications and features patients and caregivers prioritize for lung disease management.

**Methods:**

We refined a patient-facing survey based on feedback from an online focus group recruited through community and advocacy volunteer networks. The survey was then administered via ResearchMatch to uncompensated U.S. volunteers, who indicated interest in lung disease research (April 1 to May 14, 2025; 6,333 invitations). POCT perceptions were measured via a 10-point Likert scale.

**Results:**

We received 214 responses (3.4% response rate). Respondents were predominantly patients (79%), female (71%), and older than 60 years (59%). Mean ratings suggested strong perceived benefits across nine POCT benefits and low concern across most drawbacks. The greatest concern was insurance coverage for POCT-associated costs (mean score: 6.7/10). Obstructive lung conditions were most frequently prioritized for future POCT applications.

**Conclusion:**

Among a national sample of ResearchMatch volunteers affected by lung disease, interest in POCT was high, and insurance coverage remained the most salient concern. By describing preferences and concerns of patients, this study informs future design, regulatory approval, and clinical integration. These findings provide evidence for POCT developers, clinicians, and policymakers focused on improving patient-centered care.

## Introduction

The spectrum of respiratory diseases includes common upper tract respiratory infections, chronic diseases that impair gas exchange, episodes of acute airway narrowing, and life-threatening neoplastic diseases. Notably, nearly every individual in the United States will experience a respiratory illness during their lifetime. Collectively, the economic impact of respiratory diseases has been estimated at more than $170 billion annually in the United States [1].

Point-of-care technology (POCT) expedites faster, decentralized diagnosis and disease management near where a patient is receiving care. To realize POCT’s potential for managing respiratory diseases – a significant and well-known cause of morbidity, mortality, and healthcare expenditures worldwide – developers and policymakers have increasingly advocated for research studies that advance our knowledge about patient and caregiver perspectives. This research aims to address this gap by offering nationwide insights into end-user priorities and barriers to POCT adoption in lung disease. A deeper understanding of POCT user experiences may help reduce health disparities, such as improving access to timely testing in underserved, rural, or resource-limited settings.

Comparatively, little is known about how individuals who are affected by lung disease and their caregivers view devices, therapies, and support in managing respiratory conditions. The diversity, variety, and severity of lung disorders challenge our ability to assess the perspectives of patients (i.e., those with impaired gas exchange) and caregivers using a general population sample. To address this gap, we developed a targeted survey to identify which therapies, devices, and management approaches are perceived as critical by individuals who are impacted by lung disease self-care or caregiving. We refined, validated, and tested the survey using methodologies from earlier seminal studies applied in research on heart, lung, blood, and sleep disorders [2, 3].

The main aim of this research was to identify the type of POCT devices and management strategies most desired by individuals providing or receiving care for lung disease. Our focus is particularly timely, as recent healthcare system shifts toward decentralization models emphasize the importance of incorporating patients’ and caregivers’ perspectives into funding priorities, technology development decisions, and reimbursement strategies. For example, growing interest in POCT among patients and caregivers has been accompanied by increased federal support [4], including initiatives from the National Heart, Lung, and Blood Institute such as the CAPCaT program, which promotes the development of POC technologies based on end-user needs [3]. By systematically studying these perspectives, our findings aim to improve patient-centered innovation, inform reimbursement and policy decisions, and enhance clinical outcomes for those most impacted by lung conditions in the United States and worldwide.

Although POCT is frequently cited as a high-value strategy for reducing healthcare costs through earlier detection and decentralized care, its real-world effectiveness depends on patient adoption, adherence, and logistical feasibility. For instance, it has become increasingly recognized that expanded POCT use could significantly reduce hospital admissions costs [5]. To date, most POCT literature focuses on diagnostic accuracy, leaving a critical gap in understanding end-user priorities and implementation [6, 7]. Patients’ financial concerns, technological preferences, and perceived barriers, which are key determinants of whether a device is adopted or abandoned, remain underexplored [8]. This study seeks to address these gaps by identifying the POCT modalities and management options most desired by the lung disease community as well as systematically characterizing barriers to successful implementation. A roadmap for how future adoption and adherence will be measured (e.g., days of usage, user interactions) can facilitate tailored management strategies.

## Materials and methods

### Focus group population

The 2025 POCT patient-facing survey was a cross-sectional study. Prior to disseminating our survey, we conducted an online focus group session with 15 adults (age ≥18 years) to refine survey content for clarity and usability and to estimate completion time. The survey was designed to capture preferences for POCT and care–supporting devices for respiratory conditions. Participants were recruited from American Lung Association volunteers, the UMass Memorial community volunteer program, and the Conquering Diseases registry of individuals who consented to be contacted about research opportunities.

Participants completed an electronic version of the survey during the session, and we recorded completion time. After completion, participants reviewed items with study staff and rated clarity and understandability on a 3-point Likert scale. Items with at least one rating below the highest tertile were discussed and revised to improve clarity. This process emphasized usability and item clarity. We did not conduct formal psychometric validation.

### Study population

We administered the 2025 POCT patient-facing survey through ResearchMatch.org. Enrollment opened on April 1, 2025, and closed on May 14, 2025. A total of 6,333 invitations were distributed to ResearchMatch volunteers who indicated interest in lung disease research and management. A total of 214 responses were received. Inclusion criteria were age 18 years or older and able to complete the online survey independently.

### Ethics approval

The University of Massachusetts Chan Medical School Institutional Review Board determined that this study was exempt human subjects research and waived the requirement for documentation of informed consent (docket #STUDY00001679).

## Survey content, data collection, and storage

We adapted a well-tested survey instrument deployed in 2022 [9], which broadly targeted toward heart, lung, blood, and sleep disorders, and refined items to better focus on lung disease. The final survey consisted of 19 questions and was designed to take approximately 10 minutes to complete based on focus group testing. Notably, two key questions were added in 2025 to examine perspectives on POCT-relevant lung conditions and supportive technologies. Items addressing perceived benefits and concerns about POCT were assessed on a 0–10 Likert scale, where 0 indicated strong disagreement, 10 indicated strong agreement, and 5 indicated a neutral response. Demographic information including age, gender, and ethnicity was collected via multiple-choice questions with response options that included the ability to decline to answer. The responses were anonymous.

The survey interface was generated using a Research Electronic Data Capture (REDCap) data management platform. Data received from respondents were transmitted directly into the study server for storage [10–12]. The secure server is hosted by the UMass Chan network and is only accessed by authorized study personnel.

### Data analysis

Responses to questions regarding POCT characteristics, perceived benefits, and concerns were rated on a 0 to 10 scale (0 = strongly disagree, 10 = strongly agree, 5 = neutral). For each Likert scale survey item, we calculated an average response score (ARS) as the sum of individual scores normalized by the total number of responses. Categorical variables were summarized using counts and percentages. Data analysis was completed in SAS version 9.4.

## Results

### Respondent demographics

Table 1 shows a total of 214 survey responses were received (or a 3.4% response rate based on 6,333 invitations), with a completion rate of 97%. Of respondents, 151 (71%) identified as female and 58 (27%) as male. Most respondents reported their role as patients (79%), with smaller proportions identifying as informal caregivers or family members (5%), nurses (7%), physicians (2.3%), or healthcare administrators (1.4%). More than half of respondents (59%) were 60 years or older, and 38% were aged 31 to 60 years. Regarding other important demographics, most respondents (96%) were not Hispanic or Latino (Table 1). Moreover, most respondents (75%) reported never using a point-of-care device to manage lung disease, while 25% reported prior use.

**Table 1.** Demographics of survey respondents from the 2025 POCT survey^*^ Table 1 long description.

Participant demographics	Number of respondents % (*N* = 214)
**Age (Years)**	
18–30	7 (3.3%)
31–60	81 (38.0%)
More than 60 years	125 (58.7%)
**Gender**	
Female	151 (71.2%)
Male	58 (27.4%)
Other	1 (0.5%)
Prefer not to answer	2 (0.9%)
**Ethnicity**	
Hispanic or Latino	8 (3.8%)
Not Hispanic or Latino	205 (96.2%)
**Participant role**	
Patient	168 (78.9%)
Caregiver	11 (5.2%)
Nurse	14 (6.6%)
Physician	5 (2.4%)
Administrator	3 (1.4%)
Other	12 (5.6%)

* Two respondents did not complete demographic survey items.

### Lung conditions with the most need for POCT

**Figure 1.**
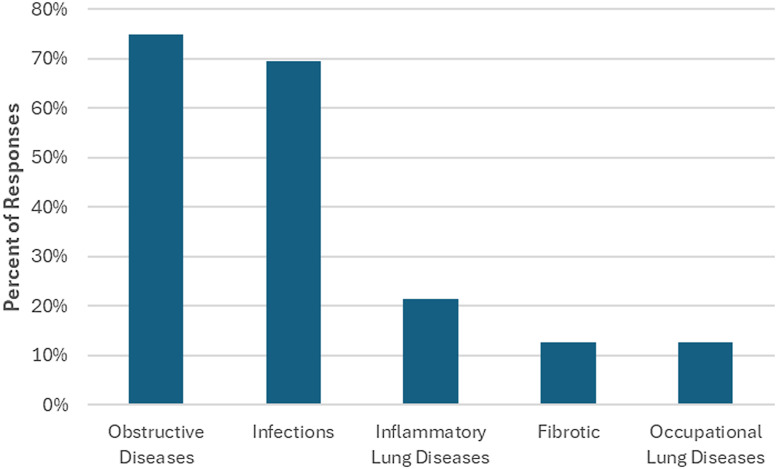
Respondents’ responses for the top three (3) lung conditions for which point-of-care tests can help monitor or manage.

Survey participants were provided with a list of lung conditions for which a point-of-care test could help them monitor or manage their condition and were asked to select and rank their top three conditions. As shown in Figure 1, obstructive diseases (e.g., asthma, COPD, cystic fibrosis) were most frequently included among respondents’ top priorities (selected by 75% of respondents). Infections (e.g., pneumonia) were the second most frequent priority (69.6%), followed by inflammatory lung diseases (21.5%). Fibrotic and occupational lung diseases were selected least frequently (12.6%).

### Preferences for new POC technology

**Figure 2.**
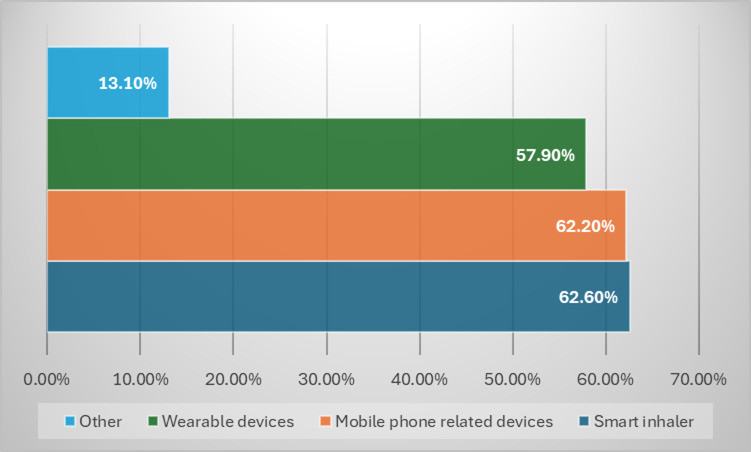
Respondents’ preferences for new POC technologies that might help them manage their lung condition(s).

Responses to the item, “What kind of new technologies might help you manage your lung condition?” (Figure 2) indicated similar interest in smart inhalers and mobile phone–connected devices (62.6% and 62.2%, respectively), followed by wearable devices such as a watch or ring (57.9%). More respondents indicated a need for technologies that support ongoing management of lung conditions (62%) than for technologies focused primarily on diagnosis (49%).

### Potential benefits of point-of-care testing

Respondents rated nine statements regarding potential benefits of POCT on a 0 to 10 scale (0 = strong disagreement, 10 = strong agreement; Table 2). Mean response scores suggested agreement with all benefit statements. The lowest mean rating was for the belief that POCT might decrease the need for taking medication (mean 6.6/10), whereas most other benefit statements had mean ratings closer to 8/10 (Table 2). Illustrative of the patient voice, one respondent shared: “With lung conditions, being able to contact my primary care provider (PCP) with data she can use to decide the next move is vital. When my breathing is compromised, there is no time for wasted calls and visits and waiting rooms and weekend hours. If we both can trust the data from a POCT, treatment is immediate. Otherwise, it’s self care and guessing till I can get to the clinic, and that can be days. No one with lung issues waits that long – we all start shooting in the dark towards a solution.”

**Table 2.** Average response scores for potential benefits of point-of-care tests Table 2 long description.

Survey Item	Average response score (ARS)
Point of care tests can provide faster results compared to traditional laboratory testing	8.2
Point of care tests can facilitate timely management decision-making	8.3
Point of care tests can improve patient satisfaction by reducing wait times	8.4
Point of care tests can enable earlier detection and intervention for better outcomes	8.6
Point of care tests can enhance patient convenience and reduce the need for multiple appointments	8.7
Point of care tests might decrease the need for taking medication	6.6
Point of care testing could improve the management of my medical condition	7.9
Point of care testing could improve patient engagement and empowerment	8.2
Point of care testing could improve overall health outcomes	8.1

Average response scores (0–10) for benefit items are shown. Higher scores indicate greater agreement with the stated benefit.

### Respondent concerns about point-of-care testing

Participants rated 12 statements regarding potential concerns about POCT on a 0 to 10 scale (0 = strong disagreement, 10 = strong agreement). Mean ratings for most concern statements were in the disagreement range, with the notable exception of insurance coverage. The item “I am concerned that my insurance might not cover the costs of the point of care test” had the highest mean score (6.7/10; Table 3). The perceived cost was the second-highest concern (mean 4.9/10).

**Table 3.** Average response scores for concerns regarding point-of-care tests Table 3 long description.

Survey Item	Average response score (ARS)
Point of care testing leads to too much testing	3.5
The accuracy of point of care testing is not good enough	4.0
The costs of point of care tests are too high	4.9
Teaching me how to use the tests may be too hard	2.0
The use of point of care testing could cause over-reliance on tests rather than my doctor’s evaluation	3.9
Point of care tests are too difficult for me to use	1.6
It would take me too long to use a point of care test	1.4
The results of point of care tests are not available quickly enough	1.9
I might have difficulty interpreting the results of a point of care test	3.0
I might not know enough about how to manage the condition to use the results of the test	3.4
The results of the test might be difficult to handle if it delivered bad news	2.7
I am concerned that my insurance might not cover the costs of the point of care test	6.7

Average response scores (0–10) for concern items are shown. Higher scores indicate greater agreement with the stated concern.

## Discussion

Our findings suggest a growing interest in POCT among the lung disease community. While patients and caregivers perceive substantial benefits in improved condition management, financial uncertainty regarding insurance coverage remains the primary barrier to adoption. These findings provide a critical roadmap for developers and policymakers to prioritize patient-centered design and transparent reimbursement pathways, ensuring that innovations in decentralized respiratory care are both accessible and effectively integrated into clinical workflows.

### The Shift towards Home-Based Monitoring and Digital Health

The high preference for mobile-integrated technologies, including smart inhalers, mobile phone–connected devices, and wearables, suggests that respondents value POCT approaches that fit naturally into daily routines and existing technology ecosystems. Mobile phone–attached spirometers and connected inhaler sensors are emerging tools that may support monitoring and self-management in obstructive lung disease [13–16]. Taken together, these findings support the view that patient-facing POCT for lung disease should prioritize seamless integration, low burden, and actionable outputs rather than stand-alone testing.

Interest in wearable monitoring devices is consistent with a growing body of work using sensors to track respiratory rate and oxygen saturation and to explore audio-based features such as cough patterns as potential markers of disease activity [17,18]. For instance, scholars acknowledge that using wearable devices to monitor health metrics, including respiratory ones, can reduce exacerbations in chronic respiratory disease [19], thereby emphasizing the value patients place on these technologies. Digital health interventions such as connected inhalers and mobile symptom tracking have shown promise in improving outcomes in chronic respiratory disease, but effectiveness in real-world settings depends on usability, sustained engagement, and integration into clinical workflows [20].

### POCT insurance coverage/reimbursement strategy

Despite high interest in POCT, cost to the healthcare system has emerged as the most prominent potential barrier to adoption [21,22]. In our survey, insurance coverage was the only concern item with a mean score in the agreement range (6.7/10), and perceived device cost was the second-highest concern (mean 4.9/10). To illustrate why insurance coverage remains a top concern, consider a hypothetical scenario: if a POCT device costs $500 annually, individuals without adequate insurance could face substantial out-of-pocket expenses, particularly impacting those on fixed incomes. This scenario highlights the need for clear and comprehensive insurance coverage and financial support mechanisms for POCT accessibility, especially among this population group.

Given that reimbursement policies for diagnostics can be complex and variable, implementation strategies for lung-related POCT should explicitly address coverage pathways and out-of-pocket costs, particularly in value-based care environments where payment often depends on demonstrated clinical utility [23]. These findings are directly relevant to payers and policymakers, especially for Medicare and Medicaid populations who may benefit most from improved access and reduced acute care utilization. To overcome these barriers, one strategy could be to advocate for clearer reimbursement guidelines that prioritize point-of-care technologies with demonstrated clinical benefits.

Additionally, partnerships between device manufacturers and insurance providers could be created to develop pilot programs that assess cost-effectiveness and simplify reimbursement processes. Establishing patient assistance programs to subsidize the costs for uninsured or underinsured individuals could also increase broader access within this community. By adopting such approaches, key stakeholders can accelerate the successful integration of POCT into clinical practice and enhance patient outcomes for users and caregivers.

### The healthcare professional’s role in POCT care

A recurring implementation question is how to balance patient autonomy with appropriate clinical oversight. Although most concern items had low mean scores, respondents expressed some concern that POCT could lead to over-reliance on tests rather than clinician evaluation (mean score: 3.9/10; Table 3). This supports positioning POCT as a supplement to, rather than a replacement for clinical assessment. Qualitative research similarly suggests that patients may value POCT as objective data while continuing to prioritize clinicians’ listening skills and examination [24].

Successful integration of POCT also depends on clinician acceptance. Prior surveys of healthcare professionals have identified concerns that expanded POCT use could complicate workflows or be perceived as challenging clinical expertise, underscoring the need for implementation models that clearly define how home-generated results are interpreted and acted upon [11].

For POCT to improve outcomes rather than add burden, users need clear and actionable guidance about how to respond to results, including escalation thresholds and when to contact clinicians. Although average concern about usability was low (e.g., mean score 2.0/10 for concerns that learning to use tests may be too hard; mean score 3.0/10 for difficulty interpreting results (see Table 3), these issues remain critical for safe implementation at scale. Practical strategies include standardized training, simple result displays, and clinician-supported action plans that translate test outputs into concrete next steps. Our data also highlight the importance of addressing implementation challenges, such as clinician buy-in, workflow integration, and support for digital literacy; these factors are essential to translating POCT from research to routine care.

### Demographic considerations in respiratory POCT adoption

The respondent population was older but expressed interest in connected technologies such as smart inhalers and wearables. Rather than assuming age is a barrier, POCT developers should plan for inclusive design that supports a range of digital literacy and physical abilities. Co-design approaches that involve older adults in interface and workflow decisions may improve activation and sustained use, as suggested by recent work in COPD populations [20].

The gender and age distribution, in general, reflects societal norms, in which older individuals and women often assume caregiving roles, potentially increasing their interest in lung disease and related technology solutions. We predict that our demographic composition may inform our understanding of technology perspectives, as it aligns with broader patterns of caregiving responsibilities.

### Study limitations

While this study provides valuable insight into POCT preferences among respondents engaged with lung disease research, several limitations should be acknowledged. First, despite our best to reach out to diverse communities, our final respondent cohort lacked racial and ethnic diversity, which we acknowledge may limit the generalizability and the ability to understand how preferences and barriers may differ across groups experiencing healthcare disparities [25]. For example, 96% of respondents identified as not Hispanic or Latino. We also did not collect data on the geographic location of respondents, so we do not know if preferences varied between rural and urban locations.

Second, as we noted earlier, the gender distribution was skewed, with 71% of respondents identifying as female and 27% as male. This imbalance may limit inference about preferences among men, who comprise a substantial portion of the lung disease population in certain conditions [26].

Third, 214 responses from 6,333 invitations correspond to a 3.4% response rate. As with many internet-based surveys, nonresponse may introduce selection bias if respondents differ systematically from nonrespondents in technology use, health status, or interest in POCT [27].

Finally, the survey relied on self-reported roles and preferences and included respondents identifying as healthcare professionals in addition to patients and informal caregivers. Future analyses should consider stratifying results by respondent role and prior POCT experience to better inform patient-facing implementation. In line with the study’s findings, we encourage follow-up studies that validate these insights across broader, more diverse populations and even those that potentially expand to international cohorts. Such efforts could further reinforce the study’s generalizability and address any regional or cultural variations in POCT adoption. Additionally, by engaging with a wider participant cohort, future research will be able to continue to adapt and refine POCT applications, ensuring their relevance and effectiveness in varied healthcare settings.

## Conclusion

This survey provides insight into perceptions of POCT among respondents engaged in lung disease self-management or caregiving. Respondents expressed interest in technologies that integrate into daily life, including connected inhalers, mobile phone–connected devices, and wearables. At the same time, potential implementation barriers were apparent. Insurance coverage was the highest-rated concern, and some respondents expressed concern about over-reliance on test results rather than clinician evaluation. These findings suggest that successful POCT adoption will depend on pairing technology with clear clinical pathways, education, and reimbursement strategies. Future work should build on these descriptive findings by evaluating preferences in more diverse populations, quantifying how preferences differ by diagnosis, role (patient versus caregiver versus clinician), and prior POCT experience, and testing implementation approaches that minimize burden while maximizing clinical actionability.

## Data Availability

We welcome open science principles and plan to share de-identified data in accordance with ethical guidelines to accelerate progress in this critical field upon reasonable request.

## Table 1. Long description

The table presents the demographics of 214 survey respondents. It has four main categories: Age, Gender, Ethnicity, and Participant role. The table contains 11 rows and 2 columns. The columns are labeled Participant demographics and Number of respondents percent (N = 214). Row 1: Age (Years), 18-30, 7 (3.3 percent). Row 2: Age (Years), 31-60, 81 (38.0 percent). Row 3: Age (Years), More than 60 years, 125 (58.7 percent). Row 4: Gender, Female, 151 (71.2 percent). Row 5: Gender, Male, 58 (27.4 percent). Row 6: Gender, Other, 1 (0.5 percent). Row 7: Gender, Prefer not to answer, 2 (0.9 percent). Row 8: Ethnicity, Hispanic or Latino, 8 (3.8 percent). Row 9: Ethnicity, Not Hispanic or Latino, 205 (96.2 percent). Row 10: Participant role, Patient, 168 (78.9 percent). Row 11: Participant role, Caregiver, 11 (5.2 percent). Row 12: Participant role, Nurse, 14 (6.6 percent). Row 13: Participant role, Physician, 5 (2.4 percent). Row 14: Participant role, Administrator, 3 (1.4 percent). Row 15: Participant role, Other, 12 (5.6 percent).

Navigate back to Table 1..

## Table 2. Long description

A table with nine rows and two columns. The columns are labeled Survey Item and Average response score (ARS). The rows list different statements about the benefits of point-of-care tests and their corresponding average response scores. Row 1: Point of care tests can provide faster results compared to traditional laboratory testing, 8.2. Row 2: Point of care tests can facilitate timely management decision-making, 8.3. Row 3: Point of care tests can improve patient satisfaction by reducing wait times, 8.4. Row 4: Point of care tests can enable earlier detection and intervention for better outcomes, 8.6. Row 5: Point of care tests can enhance patient convenience and reduce the need for multiple appointments, 8.7. Row 6: Point of care tests might decrease the need for taking medication, 6.6. Row 7: Point of care testing could improve the management of my medical condition, 7.9. Row 8: Point of care testing could improve patient engagement and empowerment, 8.2. Row 9: Point of care testing could improve overall health outcomes, 8.1.

Navigate back to Table 2..

## Table 3. Long description

A table with two columns and twelve rows. The columns are labeled Survey Item and Average response score (ARS). The rows list various concerns about point-of-care tests and their corresponding average response scores. Row 1: Point of care testing leads to too much testing, 3.5. Row 2: The accuracy of point of care testing is not good enough, 4.0. Row 3: The costs of point of care tests are too high, 4.9. Row 4: Teaching me how to use the tests may be too hard, 2.0. Row 5: The use of point of care testing could cause over-reliance on tests rather than my doctor’s evaluation, 3.9. Row 6: Point of care tests are too difficult for me to use, 1.6. Row 7: It would take me too long to use a point of care test, 1.4. Row 8: The results of point of care tests are not available quickly enough, 1.9. Row 9: I might have difficulty interpreting the results of a point of care test, 3.0. Row 10: I might not know enough about how to manage the condition to use the results of the test, 3.4. Row 11: The results of the test might be difficult to handle if it delivered bad news, 2.7. Row 12: I am concerned that my insurance might not cover the costs of the point of care test, 6.7.

Navigate back to Table 3..
